# Gibberellin‐regulated protein in Japanese apricot is an allergen cross‐reactive to Pru p 7

**DOI:** 10.1002/iid3.180

**Published:** 2017-07-06

**Authors:** Naoko Inomata, Mami Miyakawa, Michiko Aihara

**Affiliations:** ^1^ Department of Environmental Immuno‐Dermatology Yokohama City University Graduate School of Medicine 3‐9 Fukuura Kanazawa‐ku Kanagawa 236‐0004 Japan

**Keywords:** Allergen, basophil activating test, food allergy, food‐dependent exercise‐induced anaphylaxis, Japanese apricot (*Prunus mume*), peach, plum, peamaclein, gibberellin‐regulated protein, Pru m 7, Pru p 7

## Abstract

**Background:**

Gibberellin‐regulated protein (GRP, also known as peamaclein) was recently identified as a new marker allergen related to systemic reactions in peach allergy; however, its role in other fruit allergies is unknown.

**Objective:**

To investigate the allergenicity of Japanese apricot (JA) GRP and clarify the clinical characteristics of JA allergy patients with GRP sensitization.

**Methods:**

Seven patients (two males, five females, mean age 28.0 years) diagnosed with JA allergy based on relevant clinical history, positive skin test and/or challenge test were enrolled. JA GRP with a molecular weight of 6896.5 Da was purified by ion‐exchange column chromatography. We performed enzyme‐linked immunosorbent assay (ELISA), IgE‐immunoblotting, basophil activating tests (BATs), and skin prick tests (SPTs) with purified JA GRP. To investigate the cross‐reactivity between JA GRP and native Pru p 7 (nPru p 7), we performed ELISA inhibition tests. We measured specific IgE levels against apricot, peach, rPru p 1, rPru p 3, and rPru p 4 using ImmunoCAP.

**Results:**

ELISA and IgE‐immunoblotting using JA GRP showed positive reactions in six (85.7%) and seven (100%) patients, respectively. Five patients who provided informed consent for BATs and SPTs using JA GRP had positive results. In four patients who underwent ELISA inhibition tests using JA GRP and nPru p 7, IgE binding to one GRP inhibited binding to the other. The positivity for specific IgE against apricot, peach, rPru p 1, rPru p 3, and rPru p 4 was 14.3%, 57.1%, 0%, 0%, and 0%, respectively. Patients developed allergic reactions that were frequently accompanied with facial swelling, especially of the eyelids, which was dependent on cofactors, such as exercise.

**Conclusions and Clinical Relevance:**

These results indicated that GRP might be a causative allergen of JA allergy, whose onset frequently requires a cofactor, such as exercise, and might be cross‐reactive between JAs and peaches.

## Introduction

Japanese apricot (JA, *Prunus mume*) belongs to the *Rosaceae* family, which includes apricots (*Prunus armeniaca*), and is mainly harvested in Asian countries. In Japanese and Korean cooking, Japanese apricots are used in juices and sauces and as pickles. Pickled and dried Japanese apricots, umeboshi, are very popular in Japan and are eaten daily as an everyday food with steamed rice. Umeboshi has been introduced as a healthy ingredient in westernized recipes for dressings, sauces, and sushi rolls. A recent study showed that the daily consumption of JA might improve digestive dysmotility symptoms, resulting in relief from gastroesophageal reflux disease symptoms 1.

To date, few cases of JA allergy have been reported and the causative allergen is unknown 2, 3. Two proteins have been registered as apricot allergens, Pru ar 1 (Bet v 1 homolog) and Pru ar 3 (nonspecific lipid transfer protein, LTP), in the World Health Organization and International Union of Immunological Societies (WHO‐IUIS) nomenclature, but these were not derived from *Prunus mume* 4, 5, 6.

Gibberellin‐regulated protein (GRP) was first identified as the fifth peach allergen, Pru p 7 (allergen name, peamaclein), in 2013 7. We reported that Pru p 7 is a marker allergen related to systemic reactions in peach allergy patients 8. Peaches are closely related to JA by biological classification and both belong the *Rosaceae* family. Interestingly, peach allergy patients with Pru p 7 sensitization had frequent allergies caused by other fruits, especially JA 9. Therefore, we hypothesized that GRP is also contained in JA, and is cross‐reactive to Pru p 7. We identified GRP as the first allergen in JA allergy and investigated cross‐reactivity between JA GRP and Pru p 7 in JA allergy. In addition, we clarified the clinical characteristics of patients with sensitization to JA GRP.

Note: The partial sequence of amino acids in GRP, along with biochemical, clinical and immunologic data, has been submitted to the WHO‐IUIS Subcommittee for Allergen Nomenclature, which assigned the designation of Pru m 7 as a new JA allergen.

## Methods

### Patients

Seven consecutive patients diagnosed with JA allergy at the Dermatology Department of Yokohama City University Hospital in Yokohama between January 2002 and December 2012 were enrolled in the present study.

Patients were diagnosed with JA allergy if they had a convincing history of allergic reactions within 2 h after the ingestion of JA and had positive results on skin prick tests (SPTs) with JA and/or challenge tests with JA. Open food challenges using JA were performed as previously described, except in cases with a recent history within 1 year of anaphylaxis after JA ingestion and SPT positive to JA 10, 11.

In addition, we evaluated each patient's clinical history to determine the involvement of cofactors, such as nonsteroidal anti‐inflammatory drugs (NSAIDs) and exercise, which are associated with food‐dependent exercise‐induced anaphylaxis (FDEIA). The time‐period accepted for a potential relationship with a food‐allergic reaction was 2 h before food ingestion, combined with the simultaneous intake of NSAIDs 12. Regarding exercise, a 4 h period after food ingestion was established 12. FDEIA was diagnosed based on a convincing history of JA allergy, and/or positive results of challenge tests combined with exercise and/or aspirin intake.

This study was approved by our institutional review board. Written informed consent was provided by all patients.

### Preparation of Japanese apricot protein extract

JA (*Prunus mume*, cultivar Nanko strain) at the commercial unripe stage were obtained from a local grocery store. Whole JA was homogenized in a blender after the addition of an extraction solution (2 mmol/L disodium ethylenediamine tetra acetate, 10 mmol/L sodium N, N‐diethyldithiocarbamate, 3 mmol/L sodium azide, and 2% suspended solid polyvinyl polypyrrolidone at a 1:1 [*w*/*v*] ratio) 13. After filtration through gauze, the extract was centrifuged at 10,000*g* for 15 min at 4°C. After protein extraction, the samples were centrifuged and the supernatants, representing the protein extract, were collected.

### Purification of gibberellin‐regulated protein from Japanese apricot

GRP was purified from the JA protein extract by ionic exchange chromatography and membrane filtrations as described in the Supplementary Information. The protein concentration was estimated based on the molar extinction coefficient at 280 nm (6710 M/cm). The purity of protein preparations was checked by sodium dodecyl sulfate–polyacrylamide gel electrophoresis (SDS–PAGE), matrix‐assisted laser desorption/ionization‐time of flight (MALDI‐TOF) mass spectrometry and *N*‐terminal amino acid sequencing as described in the Supplementary Information.

### Purification of GRP (Pru p 7) from peaches

Native peach GRP (nPru p 7) was purified from peach pulp by ion‐exchange chromatography following as previously described 14.

### SDS–PAGE

JA crude extract (10 mg per lane) or purified JA GRP (100 ng per lane) was subjected to SDS–PAGE (12%, *w/v*) under reducing conditions using the electrophoresis system Bolt ™ (Thermo Fisher Scientific, MA, USA) according to the method of Laemmli, following the instructions supplied by the manufacturer 15. For SDS–PAGE under reducing conditions, the samples were mixed with 0.1 M Tris (pH 6.8) containing 4% (*w/v*) SDS, 20% (*w/v*) glycerol, 10% (*w/v*) 2‐mercaptoethanol, and 0.02% (*w/v*) bromophenol blue. The samples were denatured by heating at 70°C for 10 min. To ensure correct protein separation and visualization, proteins on the gel were stained with Coomassie Brilliant Blue R‐350 (Wako Pure Chemical Industries, Osaka, Japan).

### Immunoblot analysis

Proteins separated on the SDS–PAGE gel under reducing conditions were transferred onto an Immobilon‐P PVDF membrane (Merck Millipore, Bedford, MS) by wet transfer blotting methods. The membrane was incubated in 10 mM phosphate buffered saline (PBS) (pH 7.5) containing 0.1% Tween 20 and 5% skim milk for blocking. For immunoblotting using rabbit anti‐Pru p 7 peptide antibodies, antibodies were diluted 1:1000 in the same blocking buffer as for the primary antibody. Then, horseradish peroxidase‐labeled goat anti‐rabbit IgG (KPL; SeraCare, Milford, MA) was used as a secondary antibody. IgE‐immunoblotting used patient sera (1:10 dilution) in the same blocking buffer as the primary antibody. Then, horseradish peroxidase‐labeled goat anti‐human IgE (KPL; SeraCare) was used as a second antibody. Detection of the final signal was performed using an ECL Western Blotting Detection kit (GE Healthcare, Little Chalfont, UK), following the manufacturer's instructions, and recorded onto X ray films (Hyperfilm MP, GE Healthcare).

### Measurement of specific IgE to native JA GRP and Pru p 7 by enzyme‐linked immunosorbent assay

Specific IgE (sIgE) antibody to purified JA GRP was detected by enzyme‐linked immunosorbent assay (ELISA) as described in the Supporting Information 14. sIgE levels >0.91 U/mL were considered positive for native JA GRP, whereas sIgE levels>0.485 U/mL were considered positive for nPru p 7.

### ELISA inhibition assay

ELISA inhibition assays were performed using the same method as above, except that sera (1:10 dilution) were preincubated with four progressive 1:10 dilutions of the corresponding inhibitor overnight at 4°C. Cross‐reactivity between JA GRP and nPru p 7 was tested with five individual sera, using each purified allergen as a solid phase and as an inhibitor (2 μg/mL). All assays were performed in duplicate.

### Specific IgE measurements by ImmunoCAP

Serum sIgE levels (ImmunoCAP; Thermo Fisher Scientific) for apricot, peach, and recombinant allergens, rPru p 1, rPru p 3, rPru p 4, rBet v 1, and rBet v 2, were measured. According to the information provided by the manufacturer, the cutoff value of the assay was 0.35 kUA/L as class 1.

### Basophil activation test

A commercial kit (Allergenicity; Beckman Coulter, Brea, CA) was used for quantification of basophil CD203c expression as described previously 16. Briefly, heparinized whole blood was incubated with various concentrations of three types of nPru p 7 proteins at the indicated concentrations for 15 min. Anti‐IgE antibody (4 μg/mL) was used as a positive control and PBS was used as the negative control. PC7‐conjugated anti‐CD3, fluorescein isothiocyanate‐conjugated anti‐Chemoattractant receptor‐homologous molecule on Th2 cells (CRTH2), and phycoerythrin‐conjugated anti‐CD203c antibodies were also added during the reaction. The samples were analyzed on a flow cytometer. Basophils were detected based on forward side scatter characteristics and the negative expression of CD3 and positive expression of CRTH2. Upregulation of CD203c on basophils was determined using a threshold defined by the fluorescence of unstimulated cells (negative control) and expressed as CD203c^high^%. At least 500 basophils were analyzed in each assay. A sample was considered positive for basophil activation if >10% basophils were CD203c^hi^ positive and the sample induced CD203c expression to a level greater than the negative control. CD203c expression on basophils from four control subjects without JA allergy was also investigated after stimulation with JA GRP.

### Skin testing with nPru p 7

Five patients provided informed consent for additional SPT with purified JA GRP. SPTs with JA GRP (2 μg/mL in PBS) were performed and read according to a standard procedure 17. The skin was pricked with a drop of allergen from the tip of a Prick‐Lancetter (EWO CARE AB, Sweden) and the responses were read after 15 min. Histamine chloride at 10 mg/mL and vehicle (Torii Pharmaceutical Co, Tokyo, Japan) served as positive and negative controls, respectively. The elicited response of SPT was considered positive when the largest diameter of the wheal induced by the allergen was ≥3 mm 18. SPT with JA GRP was also performed on four control subjects without JA allergy.

## Results

### Demographic data of study subjects with JA allergy

Seven patients with a mean age of 28.0 (range, 14–54 years; two men and five women) were enrolled in this study (Table 1). Patient histories of other allergies demonstrated the prevalence of other food allergies was 100.0% (7/7); pollinosis, 85.7% (6/7); bronchial asthma, 14.3% (1/7); and atopic dermatitis, 28.6% (2/7). The most frequent causative food was peach (100.0%, 7/7), followed by apple (71.4%, 5/7), grape (28.6%, 2/7), strawberry, cherry, pineapple, orange, and lemon, based on the convincing history and positive SPT results with fruits. The first episode of allergic reactions to foods occurred after ingesting JA or peaches in one and six patients, respectively.

**Table 1:** Clinical characteristics of patients with Japanese apricot allergy

		Questionnaire answers: symptoms after JA ingestion				
No. of case	Age/sex	Causative food and trigger	Cofactor	Symptoms	Food allergy	Other allergic histories
1	15/F	Walking for 30 min after ingestion of umeboshi intake	Walking	Ae, Ur, Dy	JA, peach*, cherry, strawberry	AD, BA, PO
2	18/F	Walking for 30 min after umeboshi intake	Walking	Or, Ur	JA, peach*, apple, strawberry	AD, PO
3	32/F	Drinking a glass of JA juice	None	Ae, Or, La	JA, peach*, fig, grape, pineapple, orange	None
4	39/M	Ingesting umeboshi after aspirin intake	Aspirin intake	Ae, Ur, La, Na, Dy	JA, peach*	PO
5	54/F	Walking for 1 h after drinking JA juice	Walking	Ae, Na	JA, peach*, cherry fig	PO
6	14/M	Swimming for 30 min after umeboshi intake	Swimming	Ur, Dy	JA*, peach, apple, orange, pear	PO
7	24/F	Jogging for 15 min after umeboshi intake	Jogging	Ae, La, Na, Dy, Co	JA, peach*, lemon, grape	PO
	Mean age 28.0 yrs; M:F = 2:5		6/7 (85.7%)	Ae, 5; Ur, 4; Dy, 4; Or, 2; Na, 3; La, 3; Co,1	JA, 7; peach, 7; cherry, 2; apple, 2; grape, 2; orange, 2; fig, 2; strawberry, 1; pear, 1; lemon, 1; pineapple, 1	PO, 6; AD, 2; BA, 1

JA, Japanese apricot; PO, pollinosis; BA, bronchial asthma; AD, atopic dermatitis; Ae, angioedema; Ur, urticaria; Or, oral symptom; La, laryngeal tightness; Na, nasal symptom; Dy, dyspnea; Co, loss of consciousness.

* The causative food of the first episode is indicated in bold.

### Identification of gibberellin‐regulated protein as a new protein component of Japanese apricot

**Figure 1:**
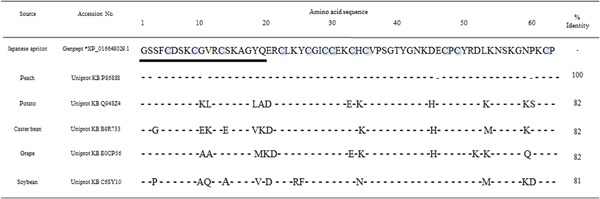
Alignment of full amino acid sequences of Japanese apricot gibberellin‐regulated protein and its homologs from other plants. The 20 *N*‐terminal underlined sequences were confirmed by protein sequencing of the isolated allergen. Twelve cysteine residues are grey‐highlighted. Gibberellin‐regulated protein residues conserved in the homologues are indicated by dashes. Sequence identities to Japanese apricot gibberellin‐regulated protein (% Id) are indicated on the right side.

SDS–PAGE of JA extract under reducing conditions revealed a 7‐kDa protein band with the sequence GSSFCDSKCGVRCSKAGYQE, which corresponded to one (accession number: XP_016648029.1) of two predicted JA GRPs registered in the GenPept database. There are no JA‐derived GRPs registered in UniProtKB (www.expasy.org). In addition, sequence similarity analysis revealed that JA GRP (accession number: XP_016648029.1) in GenPept shares 100% amino acid sequence with Pru p 7 (accession number: P86888) registered in UniProt KB (Fig. 1).

### IgE‐immunoblotting with JA GRP

**Figure 2:**
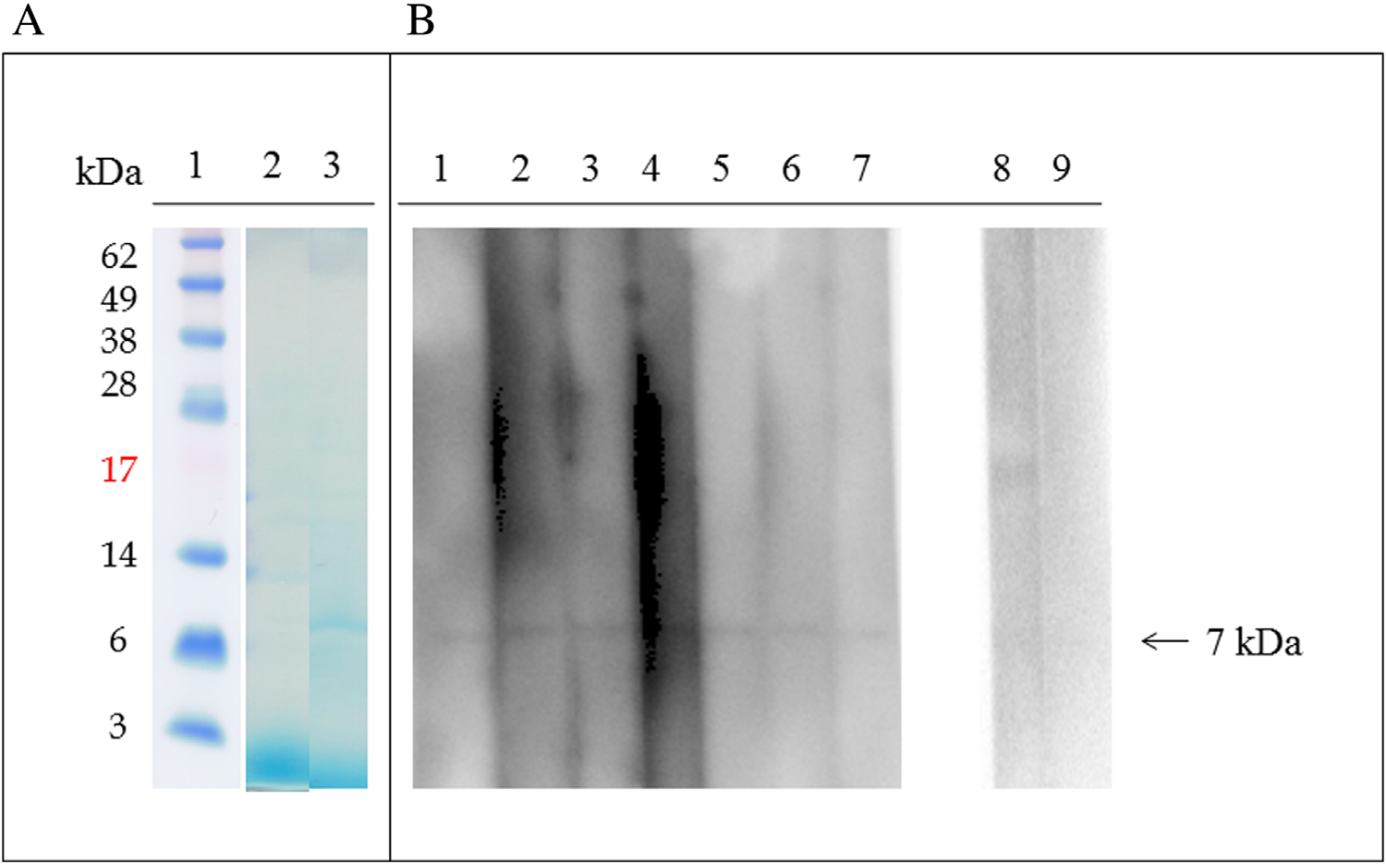
Patient IgE binding to Japanese apricot gibberellin‐regulated protein detected by IgE‐Immunoblotting. (A) Coomassie blue staining of PVDF membrane: lane 1, molecular mass standard; lane 2, Japanese apricot protein extract; lane 3, purified Japanese apricot gibberellin‐regulated protein. (B) IgE‐immunoblotting using purified Japanese apricot gibberellin regulated protein: lanes 1–7, patients 1–7; lanes 8–9, control individuals.

SDS–PAGE of the JA extract under reducing conditions showed multiple protein bands with molecular weights ranging from approximately 6 to 35 kDa. SDS‐PAGE of purified JA GRP revealed a single band with a molecular weight of 7 kDa (Fig. 2). IgE‐immunoblotting with JA GRP from seven patients, but not from three non‐JA allergic subjects, showed an IgE binding band corresponding to GRP with a molecular weight of 7 kDa.

### Measurements of specific IgE for JA GRP and Pru p 7 by ELISA

ELISA analysis showed that six of seven patients with JA allergy were positive for JA GRP (range, 0.66–121 U/mL; mean, 22.1 U/mL) (Table 2). These six patients also showed positivity for nPru p 7 (range, 0.4–4.12 U/mL, mean 1.13 U/mL). Of note, the sIgE levels for JA GRP were higher compared with nPru p 7 in these patients.

**Table 2:** Diagnostic profiles of patients with Japanese apricot allergy

		Specific IgE level													
		ImmunoCAP (UA/mL)								ELISA (U/mL)		BAT^†^(%)	SPT^‡^(mm)		Challenge
No. of case	Total IgE level (IU/mL)	Apricot	Peach	Pru p 1	rPru p 3	rPru p 4	Alder	rBet v 1	rBet v 2	JA GRP*	nPru p 7**	JA GRP	Apricot	JA GRP	JA
1	**400**	<0.35 (0)	3.40 (3)	<0.35 (0)	0.13 (0)	<0.35 (0)	<0.35 (0)	<0.35 (0)	<0.35 (0)	**121.7**	**4.12**	ND	**6.0**	ND	ND
2	**621**	**2.37 (2)**	2.09 (2)	<0.35 (0)	<0.35 (0)	<0.35 (0)	**1.09 (2)**	<0.35 (0)	<0.35 (0)	**13.0**	**0.94**	ND	**9.0**	ND	ND
3	225	<0.35 (0)	<0.34 (0)	<0.35 (0)	<0.35 (0)	<0.35 (0)	<0.35 (0)	<0.35 (0)	<0.35 (0)	**11.0**	**0.86**	**79.1**	**4.5**	**11.4**	ND
4	102	<0.35 (0)	**0.95 (2)**	<0.35 (0)	<0.35 (0)	<0.35 (0)	<0.35 (0)	<0.35 (0)	<0.35 (0)	**4.0**	**0.59**	**39.7**	**7.1**	**14.3**	**Positive** ^§^
5	54	<0.35 (0)	<0.34 (0)	<0.35 (0)	<0.35 (0)	<0.35 (0)	<0.35 (0)	<0.35 (0)	<0.35 (0)	**3.45**	**0.50**	**91.3**	**13.6**	**10.9**	ND
6	**792**	<0.35 (0)	**4.50 (3)**	<0.35 (0)	<0.35 (0)	<0.35 (0)	<0.35 (0)	<0.35 (0)	<0.35 (0)	**1.18**	**0.54**	**76.4**	**7.3**	**11.7**	ND
7	**487**	<0.35 (0)	**1.36 (2)**	<0.35 (0)	<0.35 (0)	<0.35 (0)	<0.35 (0)	<0.35 (0)	<0.35 (0)	0.66	0.40	**48.1**	**10.1**	**24.7**	ND
	Mean 387	1/7 (14.2%)	5/7 (71.4%)	0/7 (0%)	0/7 (0%)	0/7 (0%)	1/7 (14.2%)	0/7 (0%)	0/7 (0%)	6/7 (85.7%)	6/7 (85.7%)	5/5 (100%)	7/7 (100%)	5/5 (100%)	1/1 (100%)

GRP, gibberellin‐regulated protein; BAT, basophil activation test; SPT, skin prick test; ND, not done. Positivity was indicated in bold.

*Specific IgE levels >0.91 U/mL were considered positive for JA GRP.

**Specific IgE levels >0.49 U/mL were considered positive for nPru p 7.

†Dagger indicates percentage of basophil CD203c expression by JA in a basophil activation test.

‡Double dagger indicates the largest diameter of the wheal induced by the allergen.

§Patient 4 developed a severe anaphylactic reaction, accompanied with facial swelling, especially of the eyelids, wheals on his neck, oropharyngeal tightness, nasal obstruction, cough, and dyspnea accompanied by decreased arterial oxygen saturation immediately after oral challenge with 10 g umeboshi 1 hour after the intake of 1.0 g aspirin, even though he had negative results for the ingestion of umeboshi alone or aspirin intake alone.

### Basophil activation test

**Figure 3:**
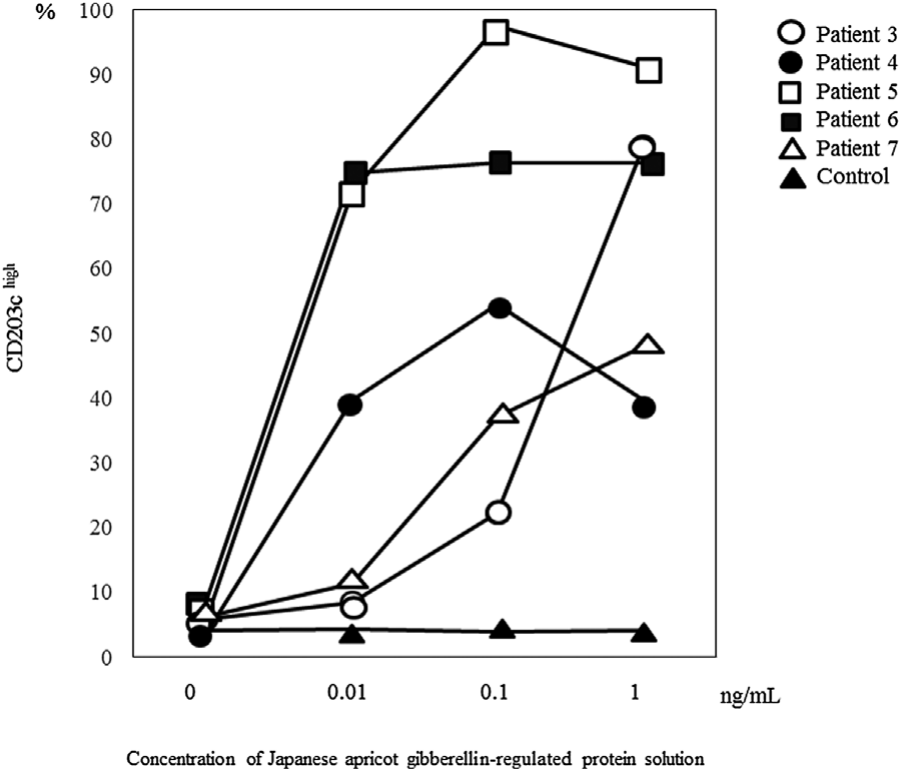
Basophil activation test with Japanese apricot gibberellin‐regulated protein. In peripheral blood mononuclear cells from all five patients (patient 3, open circles; patient 4, closed circles; patient 5, open squares; patient 6, closed squares; and patient 7, open triangles), CD203c expression was prominently induced by JA GRP, whereas the mean percentage of CD203c^high^ of four control subjects without JA allergy (closed triangles), was under 10% at all concentrations. Positive controls (anti‐IgE) showed 70.3%, 82.2%, 83.8%, 83.6%, 68.8% and 35.5% CD203c–expressing cells in patients 3–7 and control, respectively.

JA GRP induced basophil CD203c expression in a concentration‐dependent manner in all five patients who provided additional informed consent for basophil activation tests (BATs), but not in four non‐JA allergic subjects (Fig. 3, Table 2). The mean percentage of basophil CD203c expression induced by JA GRP at the highest concentration, 1 ng/mL, was 48.7% (39.7–91.3%) and 4.1% (3.4–4.9%) in JA allergy patients and four non‐JA allergic subjects, respectively.

### Skin testing

SPTs were positive for JA GRP in all five patients who provided additional informed consent for SPTs with JA GRP (Table 2). The largest wheal diameter induced by the allergen ranged from 11.4 to 24.7 mm (mean 14.6 mm). Four non‐JA allergic control subjects were negative for JA GRP by SPT.

### ELISA inhibition assays

**Figure 4:**
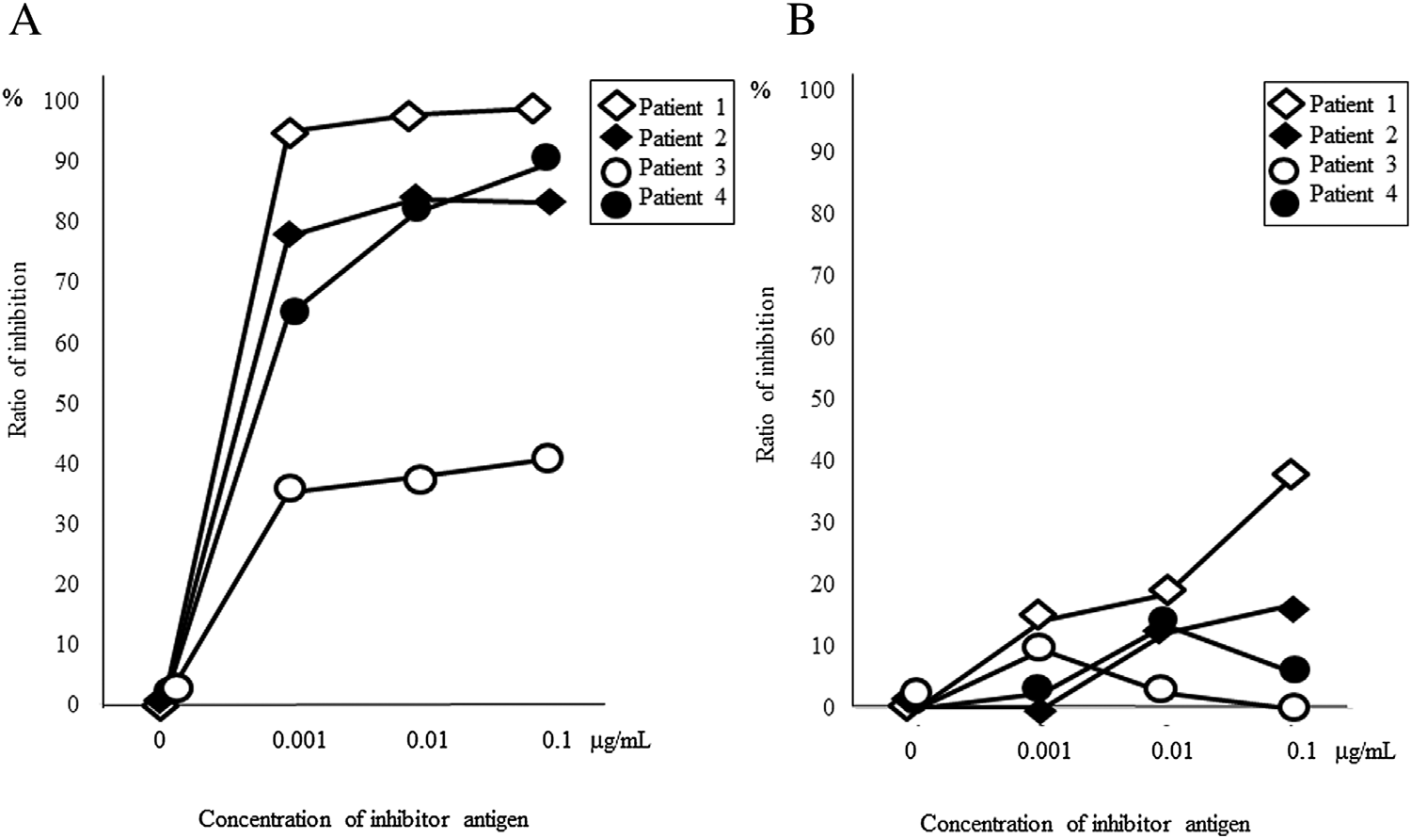
Inhibition assay by ELISA. (A) Inhibition of specific IgE binding to nPru p 7 in the solid phase with Japanese apricot (JA) gibberellin‐regulated protein (GRP). The maximum inhibition rate of four patients was 40.6–99.1%. (B) Inhibition of specific IgE binding to JA GRP in the solid phase with nPru p 7. The maximum inhibition rate of four patients was 9.57–37.2%.

To investigate potential cross‐reactivity between JA GRP and nPru p 7, ELISA inhibition tests were performed using serum from four patients who had specific JA GRP IgE levels greater than 4 U/mL (Fig. 4). IgE binding to one GRP inhibited binding to other GRP in a concentration‐dependent manner in all four patients. IgE binding to nPru p 7 was inhibited up to 99.1% by JA GRP, whereas IgE‐binding to JA GRP was inhibited up to 37.2% by nPru p 7. In all four patients, the inhibition rate of IgE‐binding to nPru p 7 by JA GRP was higher than that for JA GRP by nPru p 7. These results indicated cross‐reactivity between JA GRP and Pru p 7.

### Clinical features of JA allergy patients with JA GRP sensitization

All seven patients were allergic to JA GRP based on their positive results for JA GRP in at least two more examinations using JA GRP.

Six patients developed anaphylactic reactions. Patient 7 experienced anaphylactic shock after the ingestion of umeboshi, followed by jogging for 15 min. Interestingly, exercise and the intake of aspirin were cofactors in five patients’ self‐reports and one patient's challenge test result. Patient 4 developed a severe anaphylactic reaction, accompanied with facial swelling, especially of the eyelids, wheals on his neck, oropharyngeal tightness, nasal obstruction, cough, and dyspnea accompanied by decreased arterial oxygen saturation (SpO_2_) immediately after oral challenge with 10 g umeboshi 1 h after the intake of 1.0 g aspirin, even though he had negative results for the ingestion of umeboshi alone or aspirin intake alone. Exercise and the intake of aspirin enhanced the onset of allergic reactions in five patients (71.4%) and one patient (14.3%), respectively (Table 2). The exercise involved in episodes was jogging, swimming, or walking.

The most frequent symptom was angioedema on the face, especially the eyelids (5/7, 71.4%), followed by urticaria (4/7, 57.1%), dyspnea (4/7, 57.1%), nasal obstruction (3/7, 42.9%), laryngeal tightness (3/7, 42.9%), oropharyngeal symptoms (2/7, 28.6%), and loss of consciousness (1/7, 14.3%).

One patient (14.3%) had a positive ImmunoCAP for apricots and five patients (71.4%) showed positive results for peaches. ImmunoCAP to rPru p 1, rPru p 3 and rPru p 4 was negative in all seven patients. In addition, the rates of positive responses to ImmnoCAP against pollen from alder, rBet v 1 and rBet v 2 were 14.3% (1/7), 0% (0/7) and 0% (0/7), respectively. Total IgE concentration was 51–621 IU/mL (mean, 383, range for age: 1–300).

## Discussion

In the present study, we identified GRP as a new allergen in JA allergy. All seven patients with JA allergy were sensitized by JA GRP based on positive results of at least two further analyses (ELISA, IgE‐immunoblotting, BAT, and SPT) with JA GRP. In addition, ELISA inhibition assays using sera from JA allergy patients indicated cross‐reactivity between JA GRP and Pru p 7, suggesting that patients sensitized by GRP from JA might have cross‐reactivity to peaches and *vice versa*. Furthermore, the current study revealed that JA GRP sensitization might be related to the clinical type of FDEIA and specific clinical characteristics including eyelid edema, which appeared in approximately 70% of the patients.

GRP is a small, basic, cysteine‐rich protein with a molecular weight of approximately 6.9 kDa. GRP functions as an antimicrobial peptide and the first isolated homolog from potato, snakin‐1, was active against plant pathogens, such as bacteria and fungus 19. GRP is essential for defense against plant pathogens and therefore are conserved across a broad range of plants. Indeed, sequence similarity searches using the basic local alignment search tool (BLAST) demonstrated that protein homologs to JA GRP are present in many other plants including peaches, grapes, potatoes, castor beans, and soybeans. Interestingly, alignment of the whole amino acid sequence of one of the two predicted JA GRP (GenPept accession number: XP_016648029.1) shows 100% identity with Pru p 7 (protein accession number: P86888). MALDI‐TOF mass spectrometry demonstrated the molecular weight of purified JA GRP was 6896 Da, similar to that of Pru p 7 (6910 Da) reported previously 7.

We identified GRP as the first allergen, Pru m 7, in JA allergy. To date, few cases of JA allergy have been reported and the causative allergen was not identified 2, 3. Iijima et al. reported a patient serum IgE reacted to proteins with molecular weights of 50–60 and 75 kDa, which were not identified 3. Additionally, when we searched amino acid sequence similarities using BLAST, the apricot allergens, Pru ar 1 and Pru ar 3, showed a high identity with the peach allergens, Pru p 1 (98%) and Pru p 3 (90%), respectively, suggesting cross‐reactivity between apricot and peach proteins. We investigated the reactivity of patient serum IgE to three peach allergen components, rPru p 1, rPru p 3, and rPru p 4, using ImmunoCAP. However, all seven patients had negative ImmunoCAP results. Therefore, the major JA allergen might be GRP rather than the apricot allergens reported previously in Japan.

The present study is the first to show cross‐reactivity between GRPs involved in food allergies and derived from different fruits. ELISA inhibition tests using the respective serum of all four patients showed IgE binding inhibited IgE binding to another GRP. The cross‐reactivity between JA GRP and Pru p 7 might be explained by high similarity of their amino acid sequences. Despite the high identity of their amino acid sequences, sIgE levels against JA GRP tended to be higher than against Pru p 7. In addition, the inhibition rate of Pru p 7 by JA GRP was higher than that of JA GRP by Pru p 7. We did not determine the reason for the different inhibition rates between JA GRP and Pru p 7. However, Tuppo et al. reported that heat denaturation affects the immunological properties of Pru p 7 20. There is a possibility that during preparation process, GRPs may undergo a conformational change. In addition, there might be differences in allergen conformation, especially the epitopes, possibly caused by modifications such as protein glycosylation, even when the amino acid sequences are similar between different allergens 21. Glycosylation is a common event in protein production. Conformation or proper exposure of peptidic epitopes of glycoproteins is also frequently modulated by glycosylation mediated by intramolecular carbohydrate‐protein interactions. Therefore, the preparation process and the glycosylation of proteins might contribute to GRP antigenic properties, resulting in the different inhibition rates of allergens. Cross‐reactivity between JA GRP and Pru p 7 support the co‐existence of allergy to both JAs and peaches in our JA patients. The first episode was provoked after ingesting peaches or JAs in six and one patients, respectively. Therefore, peaches or JA might be the primary sensitizer, but it is unknown which might be more frequently the primary sensitizer.

There have been a few reports regarding the clinical features of apricot allergy, including JA. Based on clinical experiences of other *Rosaceae* family fruits, it was presumed that apricots may cause allergic reactions, ranging from mild symptoms such as oral allergy syndrome 22, 23, to severe systemic reactions, such as anaphylaxis. In the current study, JA allergy was likely to induce systemic reactions and the most frequent symptoms were facial edema, especially eyelid edema (71.4%).

Interestingly, physical exercise and/or intake of medication, such as aspirin, were required as cofactors to develop allergic reactions after the ingestion of JA in six of seven patients (84.6%). Indeed, two previous cases with JA allergy were also diagnosed with FDEIA 2, 3. In the first case, exercise was required to provoke an anaphylactic reaction after the combined allergen intake of umeboshi and wheat 2. In the second case, a challenge test combined with exercise and aspirin administration showed a positive result 3. In two of seven patients in the current study, exercise also enhanced allergic reactions after the ingestion of peach (data not shown). Thus, the onset of JA allergy might be more likely to require cofactors, especially exercise, than that of peach allergy. We hypothesized that cofactors that enhance the absorption of allergens are more likely to be required for the onset of JA allergy than peach allergy because the amount of JA consumed in one serving (approximately 1–20 g) is usually smaller than that of peaches (approximately 50–100 g). However, further studies with a larger population are needed to clarify the association between JA allergy and cofactors. The pathogenic mechanism of cofactors in food allergy remains to be clarified but might include changes in mucosal permeability induced by NSAIDs, exercise or a combination of both to enhance allergen absorption, resulting in the increased exposure of mast cells to allergens 24, 25. Anaphylaxis induced by food allergens associated with a cofactor is often described in patients allergic to ω‐5 gliadin. Anaphylactic episodes caused by plant pan–allergen LTPs, the most frequent cause of food‐induced anaphylaxis in the Mediterranean region, often occur under the influence of a cofactor, such as physical exercise and/or a medication, especially NSAIDs 12, 26. Pascal et al. reported that 32.4% of LTP‐induced anaphylaxis cases were dependent on cofactors including exercise and NSAIDs 12. However, our seven patients had negative ImmunoCAP results for rPru p 3.

Pru p 7 is stable to heating and simulated gastric digestion because of its high cysteine content (19% of total residues) similar to LTP, which forms six disulfide bridges that stabilize the small molecule 7, 27, 28. Therefore, GRP, like LTP, is suspected to be a true food allergen that has the capacity to sensitize by itself via the gastrointestinal tract and to induce severe reactions after ingestion because of its resistance to digestive enzymes. In addition, similar to Pru p 3, Pru p 7 maintains its native 3D‐structure up to 90°C but becomes unfolded at temperatures of 100–120°C, resulting in partial lack of their IgE‐binding epitopes 20. Tuppo et al. reported that only the heat‐denatured protein becomes sensitive to intestinal proteases 20. Umeboshi and JA juice, which were the causative food products in the current study, were not heated when processing the JAs. Therefore, the antigenicity of JAs might remain stable during processing of umeboshi and JA juice.

Recent reports demonstrated BATs were superior to other diagnostic tests when discriminating between peanut allergy and tolerance, particularly in difficult cases, and therefore reduced the need for oral food challenges 29. In the present study, all five patients had positive BAT results for GRP. Because GRP allergies are likely to be severe systemic reactions, BAT might be a useful diagnostic tool for screening GRP allergies to reduce the requirement for oral food challenges and to make correct diagnoses.

This study had some limitations including small number of subjects enrolled. To date, only a few cases of JA allergy have been reported. JA allergy might be underestimated because of the low reproductivity of allergic reactions in a cofactor‐dependent manner. In addition, information is available on GRP derived from JA but not from apricots in all databases. Therefore, determining the clinical features and allergens of JA allergy might help diagnose patients with allergy to JAs and apricots. However, further investigation of a large number of subjects is required to clarify whether GRP sensitization might be involved in JA allergy provoked by cofactors.

In conclusion, our results indicated that GRP is a cross‐reactive allergen between JA and peaches, and might be a causative allergen of JA allergy, especially FDEIA. If patients are suspected to have JA allergy, the possibility of cofactor involvement in anaphylactic reactions caused by JA and cross‐reactivity should be considered.

## Conflict of Interest

All authors have no financial supports or relationships that may pose a conflict of interest.

## Supporting information

Additional supporting information may be found in the online version of this article at the publisher's web‐site.

**Figure S1**. Purity of Japanese apricot (JA) and reactivity of polyclonal antibodies to Pru p 7 and Pru p 3 peptides. (A) SDS–PAGE of JA extract, 10 μg/lane (lane 1) and the purified JA gibberellin‐regulated protein, 10 ng/lane (lane 2). (B) Separation of proteins in JA extract, 10 ng/lane (lanes 1 and 2) and the purified JA gibberellin‐regulated protein, 10 ng/lane (lanes 3 and 4). Proteins were electro‐transferred to PVDF membranes and incubated with anti‐Pru p 7 peptide antibodies (lanes 1 and 3) or anti‐LTP peptide Ab (lanes 1 and 3).

**Figure S2**. Mass spectrometry measurements. Annotated mass spectrum of Japanese apricot gibberellin‐regulated protein, indicating an average mass of 6896.5 Da.

Supporting Information S1.
