# Supplementary figures and images for: Gibberellin‐regulated protein in Japanese apricot is an allergen cross‐reactive to Pru p 7
Source: Immun Inflamm Dis. 2017 Jul 6;5(4):469–79. doi: 10.1002/iid3.180 (PMC5691307; doi:10.1002/iid3.180)

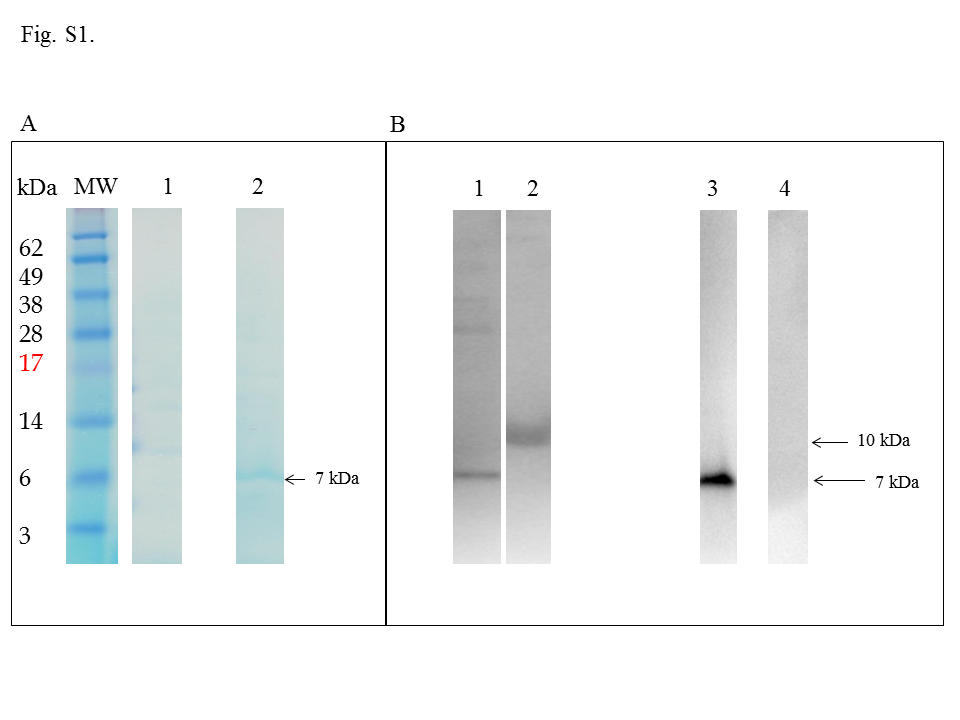

Supplement: Supplementary file 1 — Figure S1. Purity of Japanese apricot (JA) and reactivity of polyclonal antibodies to Pru p 7 and Pru p 3 peptides. (A) SDS–PAGE of JA extract, 10 μg/lane (lane 1) and the purified JA gibberellin‐regulated protein, 10 ng/lane (lane 2). (B) Separation of proteins in JA extract, 10 ng/lane (lanes 1 and 2) and the purified JA gibberellin‐regulated protein, 10 ng/lane (lanes 3 and 4). Proteins were electro‐transferred to PVDF membranes and incubated with anti‐Pru p 7 peptide antibodies (lanes 1 and 3) or anti‐LTP peptide Ab (lanes 1 and 3). [file IID3-5-469-s001.tif]

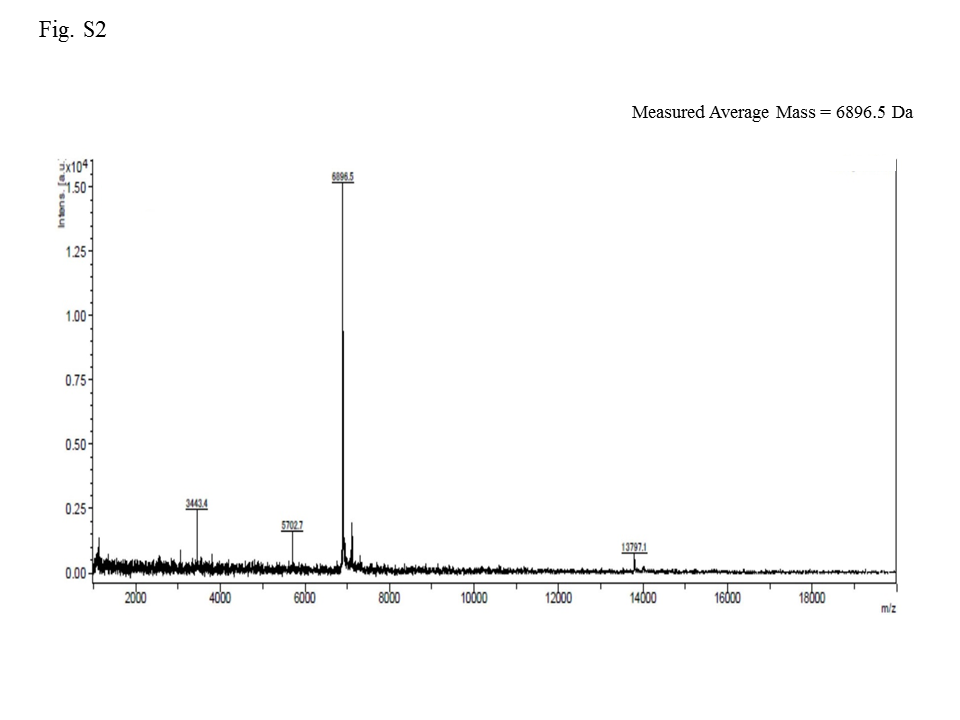

Supplement: Supplementary file 2 — Figure S2. Mass spectrometry measurements. Annotated mass spectrum of Japanese apricot gibberellin‐regulated protein, indicating an average mass of 6896.5 Da. [file IID3-5-469-s002.tif]
