# Supplementary material for: Gibberellin‐regulated protein in Japanese apricot is an allergen cross‐reactive to Pru p 7
Source: Immun Inflamm Dis. 2017 Jul 6;5(4):469–79. doi: 10.1002/iid3.180 (PMC5691307; doi:10.1002/iid3.180)
Supplement: Supplementary file 3 — Supporting Information S1. [file IID3-5-469-s003.docx]

**Supporting Information**

Title: Gibberellin-regulated protein in Japanese apricot is an allergen cross-reactive to Pru p 7

**METHODS**

*Estimation of the protein concentration*

Protein concentrations were quantified using a Qubit 2.0 fluorometer (Thermo Fisher Scientific, Waltham, MA, USA) according to the information provided by the manufacturer.

*Synthesis of polyclonal antibodies specific to Pru p 7 and Pru p 3 peptides*

Based on the amino acid sequence of Pru p 7 and Pru p 3 (the UniProt Knowledgebase accession No. P86888 and P81402), the individual peptides were synthesized by solid-phase peptide synthesis using Fmoc chemistry. This form was developed to show the basic steps in solid phase peptide synthesis using Fmoc chemistry as described previously [1]. The purity of peptides was <99.23% by high performance liquid chromatography (HPLC). To ensure that peptides induced an immune response and produced antibodies, ECPCYRDLKNSKGN originated from gibberellin-regulated protein (GRP) and CCNGCRNVNNLART originated from lipid transfer protein (LTP), were conjugated to keyhole limpet hemocyanin (KLH) as a carrier protein. Three rabbits (Nippon SLC Co., Shizuoka, Japan) were immunized by four subcutaneous injection of 25 µg of individual BSA-conjugated peptides emulsified in Freund’s complete adjuvant, 20 µg of Freund’s incomplete adjuvant, and 10 µg of 10 mM phosphate buffer saline (PBS) (pH 7.4) containing 0.15 M NaCl at 2-week intervals. On the 14^th^ day after the final injection, antiserum was collected from rabbits.

*Immunoblot analysis*

To demonstrate the lack of contamination of Japanese apricot (JA) LTP in the purified JA GRP, we performed immunoblotting using polyclonal antibodies specific to Pru p 7 and Pru p 3 peptides.

Purified JA crude extract and JA protein were subjected to sodium dodecyl sulfate–polyacrylamide gel electrophoresis (SDS-PAGE) (12% (*w/v*), 10 ng per lane) under reducing conditions using the electrophoresis system Bolt™ (Thermo Fisher Scientific) according to the method of Laemmli, following the instructions provided by the manufacturer [2]. Proteins separated by the SDS-PAGE under reducing conditions were transferred onto an Immobilon-P polyvinylidene difluoride (PVDF) membrane (Merck Millipore, Bedford, MA, USA) by wet transfer blotting methods. The membrane was incubated in 10 mM PBS (pH 7.5) containing 0.1% Tween 20 and 5% skim milk for blocking. The membrane was incubated for 1 hour at room temperature with the allergen specific antibodies indicated in PBS/0.05% Tween 20. Bound primary antibodies were detected by horseradish peroxidase-conjugated goat anti-mouse IgG (Promega, Tokyo, Japan) and an ECL Western blotting detection kit (GE Healthcare, Little Chalfont, UK), following the manufacturer’s instructions. The obtained chemiluminescence signals were detected on X-ray films (Hyperfilm MP, GE Healthcare).

*N-terminal amino acid sequence analysis*

The single band in SDS-PAGE using the JA fraction containing the 7-kDa protein, which bound to anti-Pru p 7 peptide antibodies but not to anti-Pru p 3 peptide antibodies in IgG-immunoblotting, under reducing conditions were electroblotted onto a PVDF membrane as described above. After staining with Coomassie Brilliant Blue, the protein band corresponding to 7 kDa was excised and analyzed on a Procise 491 cLC protein sequencer (Applied Biosystems, Foster City, CA, USA), following the manufacturer’s recommendations. Twenty *N*-terminal amino acids were determined for the band. The obtained sequences were analyzed by the Phytozome and BLAST programs to identify proteins in the data bases.

*Matrix-assisted laser desorption/ionization-time of flight (MALDI-TOF) mass spectrometry*

The purity of the proteins was tested and the molecular mass of the purified peach proteins was estimated by MALDI-TOF mass spectrometry measurements performed on an UltrafleXtreme™ (Bruker, Billerica, US).

*Puriﬁcation of GRP from JA*

JA GRP was purified from JA pulp protein extract by ion-exchange column chromatography as previously described with some modifications [3,4]. JA (*Prunus mume*, cultivar Nanko strain) at the commercial unripe stage were obtained from a local store. Whole JA was homogenized with an extraction solution (2 mmol/L disodium ethylenediamine tetra acetate, 10 mmol/L sodium N, N-diethyldithiocarbamate, 3 mmol/L sodium azide, and 2% suspended solid polyvinyl polypyrrolidone at a 1:1 [*w/v*] ratio) [5]. A method based on ion-exchange column chromatography for the enrichment of fruit-allergenic extracts, as described by Martinez et al., was used.^5^ After filtration through gauze, the extract was centrifuged at 10,000 ×g for 15 min at 4°C. Cation exchange resin (Sepharose Fast Flow, GE Healthcare, Little Chalfont, UK) was added to the supernatant and mixed overnight at 4°C. The resin was collected by centrifugation, packed and washed with 20 mmol/L phosphate buffer at pH 5.0 in a column. The proteins absorbed into the resin were eluted with 0.5 mol/L NaCl in the same buffer. The eluate was monitored at 280 nm. The separated fractions were manually collected and analyzed.

To eliminate proteins with a molecular weight greater than 10 kDa, the sample was filtrated using Ultracel 10K Amicon Ultra filters (Merck Millipore,). The sample was concentrated by ultrafiltration through Ultracel 3K Amicon Ultra filters (Merck Millipore). Further purification was achieved by ion-exchange column chromatography on a cation exchanger Sepharose Fast Flow (GE Healthcare), eluting in a stepwise gradient from 0% to 100% of a 0.2M NaCl, 20 mM acetate buffer.

Fractions containing the protein were concentrated and buffer exchanged to PBS by ultrafiltration on an Ultracel 3K Amicon Ultra filters as above. The purified JA GRP was sterilized by filtration through a 0.22-μm pore diameter membrane (Merck Millipore) and frozen at −80°C.

*Measurement of specific IgE specific to JA GRP and Pru p 7 by ELISA*

Specific IgE (sIgE) antibody to purified native Pru p 7 (nPru p 7) was detected by ELISA as previously reported [4]. The wells of a SUMILON® multi-well, flat bottom 96-well plate (Sumitomo Bakelite Co., Tokyo, Japan) were coated with purified JA GRP or nPru p 7 (2 µg/mL in PBS) at 50 µL/well. The plate was sealed and left overnight at 4°C. Then it was blocked with PBS containing 1% skim milk and 0.1% Tween 20 (PBS-T) for 1 hour at room temperature, after which 50 μL of the patient sera diluted to 20% in 1% skim milk PBS-T was added to the wells, followed by incubation for 1 hour at room temperature. The plate was then washed with 1% skim milk PBS-T. A total of 50 μL of 0.1 μg/mL anti-human IgE-horseradish peroxidase conjugate (KPL, Gaithersburg, MD, USA) in 1% skim milk PBS-T was added to the wells, and the plate was incubated for 1 hour at room temperature. The plate was washed, and the colorimetric reaction was developed by adding 1-Step Ultra TMB-ELISA (Thermo Fisher Scientific) and incubated for 15 min at room temperature. The reaction was stopped by adding 2 M H_2_SO_4_. Absorbance at 450 nm was read with a Multiskan™ GO Microplate Spectrophotometer (Thermo Fisher Scientific). Absorbance was converted into “unit values” based on a standard curve, which was made using house dust mite, *Dermatophagoides farinae* (*Der f*) crude extract (Life Science Laboratories, Inc., NY, US) and pooled serum containing IgE specific to Der f 1, whose titers were calculated by ImmunoCAP (Thermo Fisher Scientific). Serum that contained IgE specific to *Der f* (1686 UA/mL) from three patients were chosen as standards. Serial dilution was performed using 1% skim milk PBS-T, starting at 20 times dilution, with subsequent doubling of the dilution factor up to 100,240-times dilution. To create a curve for the conversion of absorbance values to “unit” values, the absorbance of the 20 times-diluted serum was defined as that corresponding to 84.3 units, with the absorbance of the 40-times-diluted serum as 42.15 units, that of the 640-times-diluted serum as 2.63 units, and so forth, such that the absorbance at each dilution factor corresponded to a “unit” value. Control sera from three non-atopic subjects without food allergies were used as negative controls. All tests were performed in duplicate.

Specific IgE levels >0.91 U/mL (mean unit ± 3 SD of the negative control value) were considered positive for native JA GRP, whereas specific IgE levels> 0.485 U/mL (mean unit ± 10 SD of the negative control value) were considered positive for nPru p 7.

*Preparation of JA GRP solution for skin testing*

JA GRP was solubilized in PBS and sterilized by membrane filtration through a 0.22-μm filter (Merck Millipore), in a sterile horizontal laminar flow hood. The final protein concentration was 2 μg/mL.

**Results**

*Analysis of the purity of JA GRP by immunoblotting using anti-Pru p 3 and Pru p 7 peptide antibodies*

Rabbit anti-Pru p 7 peptide antibodies bound to protein with a molecular weight of 7 kDa in the JA protein extract and the purified JA GRP, but not to a protein with a molecular weight of 10 kDa in any of the extracts (Fig. S1). Rabbit anti-Pru p 3 peptide antibodies bound to the protein with a molecular weight of 10 kDa in the JA protein extract, but not to the protein with a molecular weight of 7 kDa in the purified JA GRP extracts. These results indicated no contamination of JA LTP in the purified JA GRP.

*Mass spectrometry*

The average masses of purified JA GRP were 6896.5 Da by MALDI-TOF mass spectrometry (Fig. S2), which was similar to that of Pru p 7 (6910.8 Da) registered in the UniProt Knowledgebase.

**Figure legends**

Fig. S1. Purity of Japanese apricot (JA) and reactivity of polyclonal antibodies to Pru p 7 and Pru p 3 peptides. (A) SDS-PAGE of JA extract, 10 μg/lane (*lane 1*) and the purified JA gibberellin-regulated protein, 10 ng/lane (*lane 2*). (B) Separation of proteins in JA extract, 10 μg/lane (*lanes 1 and 2*) and the purified JA gibberellin-regulated protein, 10 ng/lane (*lanes 3 and 4*). Proteins were electro-transferred to PVDF membranes and incubated with anti-Pru p 7 peptide antibodies (*lanes 1 and 3*) or anti-LTP peptide Ab (*lanes 1 and 3*).

Fig. S2. Mass spectrometry measurements. Annotated mass spectrum of Japanese apricot gibberellin-regulated protein, indicating an average mass of 6896.5 Da.

REFERENCES

1. Sheppard R. The fluorenylmethoxycarbonyl group in solid phase synthesis. *J Pept Sci* 2003; **9**: 545-552.
2. Laemmli UK. [Cleavage of structural proteins during the assembly of the head of bacteriophage T4.](http://www.ncbi.nlm.nih.gov/pubmed/5432063) *Nature* 1970; **227**:680-685.
3. Tuppo L, Alessandri C, Pomponi D *et al*. [Peamaclein - a new peach allergenic protein: similarities, differences and misleading features compared to Pru p 3.](http://www.ncbi.nlm.nih.gov/pubmed/23278887) *Clin Exp Allergy* 2013; **43**: 128-140.
4. Inomata N, Miyakawa M, Aihara M. [Eyelid edema as a predictive factor for sensitization to Pru p 7 in peach allergy.](http://www.ncbi.nlm.nih.gov/pubmed/26916754) *J Dermatol* 2016; **43**:900-905.
5. Björkstén F, Halmepuro L, Hannuksela M, Lahti A. Extraction and properties of apple allergens. *Allergy* 1980; **35**, 671-677.
